# Integrative biclustering of heterogeneous datasets using a Bayesian nonparametric model with application to chemogenomics

**DOI:** 10.1186/1471-2105-12-S7-A6

**Published:** 2011-08-05

**Authors:** Dazhuo Li, Eric C Rouchka

**Affiliations:** 1 Department of Computer Engineering and Computer Science, University of Louisville, Louisville, KY, 40292, USA

## Motivation

The identification of protein function and the prediction of ligand-target interaction is an active research field that is facilitated by means of categorizing ligands and proteins into biologically sensible groups. Because of the pharmacological fact that related drugs can bind to receptors without obvious sequence or structural similarity, it is appropriate to categorize proteins based not only on their sequence or structures but also on the chemical structure and the phenotypic side-effect of their ligands. In chemogenomic studies where the complete set of ligands for a protein is not known *a priori*, integrating the *de novo* detection of interacting ligand and protein groups into the categorization process can guide the process towards more biologically sensible solutions.

## Results

We present the Weighted Infinite Relational Model (WIRM) that jointly detects biologically sensible ligand groups and protein groups by integrating the clustering of various data types including chemical compound descriptors, protein sequences, ligand-target bindings and pharmaceutical effects. WIRM takes advantage of the Bayesian nonparametric paradigm for integrating multiple data types, for allowing for missing values (e.g. unknown ligand-target interaction) in the data, for automatically inferring the number of clusters without explicit model comparison, and for predicting the ligand-target interactions. Because some of these data types, to varying degrees, may suggest relationships having no implication for ligand-target interactions or for biological sensible ligand and protein groups, WIRM allows different types of data to have different weights based on prior knowledge of their quality or relevance.

## Conclusion

We apply WIRM to the ion channel proteins and G-protein-coupled receptors. We validate its performance using functional annotation and ligand-target interaction. We also test the relationship among multiple data types by varying the weights which indicate the impact of each data type on the model. The categories and interactions inferred by WIRM both confirm known biology and suggest novel predictions.

